# Study on Near-Net Forming Technology for Stepped Shaft by Cross-Wedge Rolling Based on Variable Cone Angle Billets

**DOI:** 10.3390/ma11081278

**Published:** 2018-07-25

**Authors:** Sutao Han, Xuedao Shu, Chang Shu

**Affiliations:** 1 Faculty of Mechanical Engineering & Mechanics, Ningbo University, Ningbo 315211, China; hansutao@foxmail.com; 2 Department of Mechanical Engineering, Northwestern University, Evanston, IL 60208, USA; 3 Department of Mechanical and Aerospace Engineering, University of Florida, Gainesville, FL 32608, USA; changshu@ufl.edu

**Keywords:** stepped shaft, cross wedge rolling, plastic flow kinetic theories, variable cone angle billets, near-net forming

## Abstract

Considering problems about concaves at the stepped shaft ends, this paper established the plastic flow kinetic theories about metal deforming during the cross-wedge rolling (CWR) process. By means of the DEFORM-3D finite element software and the point tracing method, the forming process of stepped shafts and the forming mechanism of concaves at shaft ends were studied. Based on the forming features of stepped shafts, rolling pieces were designed using variable cone angle billets. Single-factor tests were conducted to analyze the influence law of the shape parameters of billet with variable cone angle on end concaves, and rolling experiments were performed for verification. According to the results, during the rolling process of stepped shafts, concaves will come into being in stages, and the increasing tendency of its depth is due to the wave mode, the parameters of cone angle **α**, the first cone section length **n**. Furthermore, the total cone section length **m** has an increasingly weaker influence on the end concaves. Specifically, cone angle **α** has the most significant influence on the quality of shaft ends, which is about twice the influence of the total cone section length **m**. The concave depth will decrease at the beginning, and then increase with the increasing of the cone angle **α** and the first cone section length **n**, and it will decrease with the increasing of the total cone section length **m**. Finite element numerical analysis results are perfectly consistent with experimental results, with the error ratio being lower than 5%. The results provide a reliable theoretical basis for effectively disposing of end concave problems during CWR, rationally confirming the shape parameters of billets with a variable cone angle, improving the quality of stepped shaft ends, and realizing the near-net forming process of cross-wedge rolling without a stub bar.

## 1. Introduction

The process of cross-wedge rolling (CWR) plays a vital role in the thermoplastic forming of steels thanks to its many advantages including high efficiency, material saving, energy saving, and environmental protection [1,2]. In the process of CWR, plastic deformation including axial extension and radial compression occurs on rolling pieces. As the flow velocity of surface metal is faster than that of core metal, in order to ensure the quality of rolling ends, stub bars of a certain length (about 8–10% the mass of the rolling piece), together with sufficient machining allowance, need to be reserved. As a result, the utilization of material is usually lower than 85% during the CWR process, and this turns out to be the bottleneck of CWR development, and cannot meet the requirements of the conservation-minded society [3,4,5]. 

Considering the stub bars generated in the CWR process, research studies has been done by experts all over the world from different perspectives. Among them, Danno and Awano [6] studied the influence laws of different process parameters on the volume of stub bars formed during CWR. Pater [7,8] obtained the forming laws of concaves by analyzing the stress–strain and metal flow during CWR, and proposed to erect clamps on rolling ends to control their quality. In China, Wei Xinhong [9] illustrated the formation mechanism of end concaves from different perspectives using the displacement method, grid method, stress and strain analysis, etc. Zhenhai Ma [10] studied the influence laws of process parameters on the volume of stub bars, and put forward the theory that the concave size would decrease with the increasing of the forming angle and the constant size of the rolling piece’s end, and would increase with the increasing of the spreading angle and deformation quantity. Wangming Zu and Xinhong Wei [11,12] erected side wedges on the die in order to restrain the flow of the surface metal at the rolling end, thus decreasing the size of the concave. Wenwei Gong [13] put forward the method to design press blocks on the die in order to restrain the flow of the surface metal of the rolling piece, thus constraining the formation of concaves. Chuan Liu and Jie Wei [14,15] designed a closed–open cross-wedge rolling die in order to change the metal flow direction of rolling piece, and thus enhancing the quality at shaft ends. Refering to Pater’s cross-wedge rolling technology based on chamfered end billets [16], Xiaoying Zhang and Bin Hu [17,18], by analyzing the reasons of end concave formation, put forward the method of changing the shape of billet ends to decrease concave size. However, stepped shafts that have been widely applied are not covered in the above research studies, which were only limited to the rolling of simple shafts such as oil pump shafts, and research on the shaft end quality forming with conical billets has not been deepened. The research work of the above experts provides a reference for the research ideas and methods of the influence of the billet shape on the end quality.

This paper, by means of the DEFORM-3D 11.0 finite element software, established the finite element model of stepped shaft rolling. Based on the study on the forming mechanism of stepped shaft rolling, the method to design billet ends to the shape with a variable cone angle was raised and aimed at the staged deformation of stepped shafts during CWR, for the control of shaft end quality. Single-factor tests were conducted to study the influence laws of cone parameters on the quality of stepped shaft ends. In combination with rolling experiments for verification, it illustrated that billets with a variable cone angle could effectively reduce the size of stub bars at the rolling end. The results provide a reliable theoretical basis for effectively disposing of end concave problems during CWR, rationally confirming the shape parameters of billets with variable cone angles and improving the quality of stepped shaft ends.

## 2. Materials and Methods

### 2.1. Plastic Flow Kinetic Theories of Metal Deformation in CWR 

**Figure 1:**
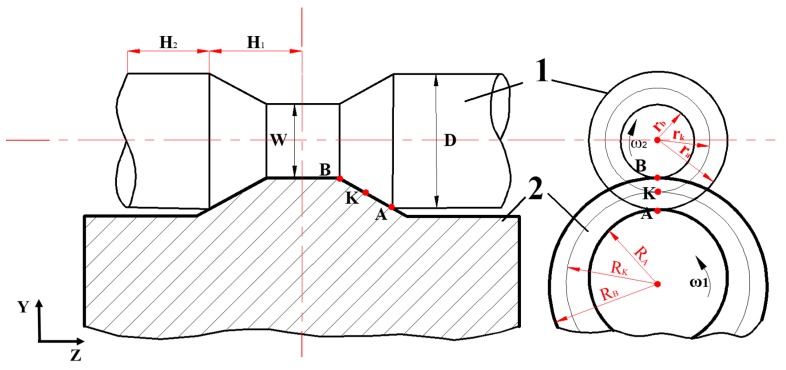
The front view and side view of the typical cross-wedge rolling (CWR) process. 1 is the rolling piece, 2 is the roller.

The CWR process is as illustrated in Figure 1. The rolling piece rotates clockwise under the driving of the roller that rotates anticlockwise. At the contact surface with the rolling piece, the roller has the minimum circular velocity at point **A** and the maximum circular velocity at point **B**, which is located on the wedge top of the die. At point **K**, the roller and the rolling piece have identical velocity. In the figure, $\omega_{1}$ refers to the rotational angular velocity of the roller, $\omega_{2}$ is the rotational angular velocity of the rolling piece, $V_{K}$ is the linear velocity of the roller die at point **K**, R_K_ is the rolling radius, which is defined by the distance from point **K** to the rotation center of the roller, **H**_1_ is the length of the rolled section, and **H**_2_ is the length of the unrolled section. The rolling piece is fed along the die and the rotational angular velocity of the rolling piece is smaller than its velocity at the rigid contact with the wedge of die. The relationship between the rotational angular velocity of the rolled piece and the linear velocity of the wedge [1] is determined by the following parameters:

(1) $$\omega_{2}=\frac{V_{K}}{r_{K}}\;$$

With increasing the length of the rolled section, the length of unrolled section will decrease. Based on the incompressibility of metallic bulk and the stable process of rolling, the below formula is obtained:

(2) $$\delta =\frac{D}{W}\;$$

(3) $$\Delta H_{1}=\Delta H_{2}\delta^{2}\;$$

wherein **ΔH_1_** is the increment of the rolled section, **ΔH_2_** is the increment of the unrolled section, *δ* is the compression ratio, D is the original diameter of the rolling piece, and W is the diameter after rolling.

**Figure 2:**
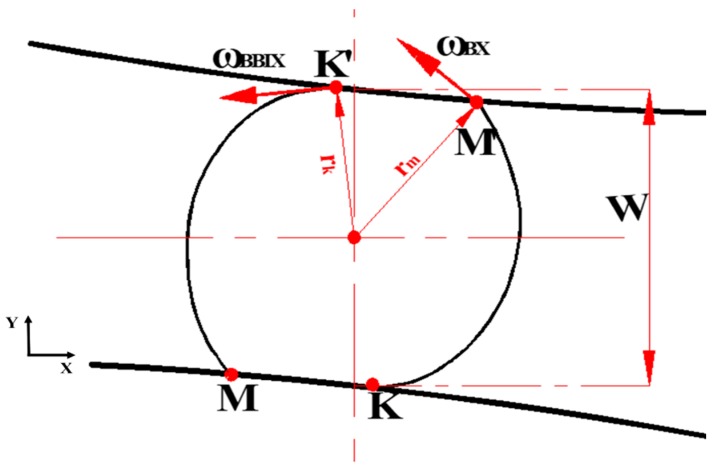
Schematic diagram deformation of simple shafts under load during CWR.

Due to the forming wedge pressure, metal in the rolled section would deform into an oval shape. The rotational angular velocity of the rolling piece increased gradually from the inlet to the outlet of the deformation area. As a result, fiber crimp occurred at the axial direction on the rolling piece. In this paper, metallic particles were regarded as the analysis objects, to study the displacement velocity of metal in the rolling area. As shown in Figure 2, the transient variable of the velocity of metallic particles at the direction of axis OX is:

(4) $$\Delta V_{x}=(\omega_{BBIX}-\omega_{BX})r_{b}\;$$

The displacement velocity at axis OZ is:

(5) $$\Delta V_{Z}=(V_{HZ}-V_{Z})\tan\beta =V_{\mu H}\tan\beta \;$$

where in $\omega_{\mathrm{BBIX}}$ is the angular velocity at the outlet of the deformation area of the rolling piece, $\omega_{\mathrm{BX}}$ is the angular velocity at the inlet of the deformation area of the rolling piece, $r_{b}$ is the radius of rolling piece at the shaping completion area, $V_{HZ}$ is the axial velocity of spreading of the wedge, $V_{Z}$ is the axial velocity of extension of the rolling piece, $V_{\mu H}$ is the sliding velocity, and β is the spreading angle. 

By means of Formulas (4) and (5), it is able to easily obtain the inclination angle between the crimped metal fiber of the rolling piece and the vertical axis:

(6) $$\varphi =\arctan\frac{\Delta V_{X}}{\Delta V_{Z}}=\arctan\frac{(\omega_{\mathrm{BBIX}}-\omega_{\mathrm{BX}})d}{2V_{\mu H}\tan\beta }\;$$

**Figure 3:**
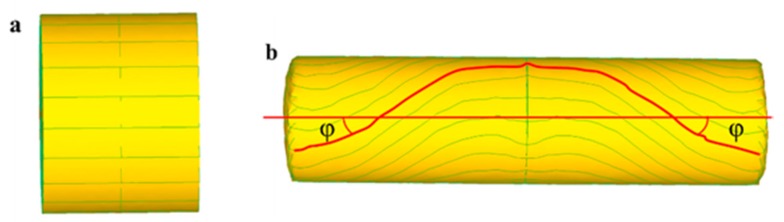
Results of finite element simulation of metal crimping of simple shaft: (**a**) billet; (**b**) rolled piece.

Due to ignoring the difference between the rolling velocity in the rolling area and the velocity of metal deformation, as well as the longitude curve of metal deformation during calculation, the crimping angle in the rolling experiment is relatively larger. The results of finite element simulation are as shown in Figure 3. 

As for metal in the unrolled section, which is the metal at the end face, the displacement velocity at the stable rolling stage is composed of two elements, including the circular motion in the cross-section and the linear motion at the axial direction. See the formula below:

(7) $$\begin{array}{l} x=a\cos\omega_{2}t\\ y=a\sin\omega_{2}t\\ z=bt \end{array}\;$$

Wherein a is the radial distance from the metallic particle to the axis of the rolling piece. 

At the spreading stage of the rolling process, the rotary radius of the rolling piece and the geometrical shape of the deformation area did not change any more, and spreading length became the exclusive element of change. At this moment, rolling and shaping became stable. Considering the incompressibility of deforming metal, the displacement curve of the end metal at the stable rolling stage is expressed as follows:

(8) $$\begin{array}{l} x=a\cos\omega_{2}t\\ y=a\sin\omega_{2}t\\ z=\frac{{\left(r_{0}+r_{1}\right)}^{3}\left(r_{0}-r_{1}\right)\sin\beta \tan\beta }{8r_{0}^{2}\tan\alpha }t \end{array}\;$$

### 2.2. Confirmation of the Finite Element Simulation Scheme 

**Figure 4:**
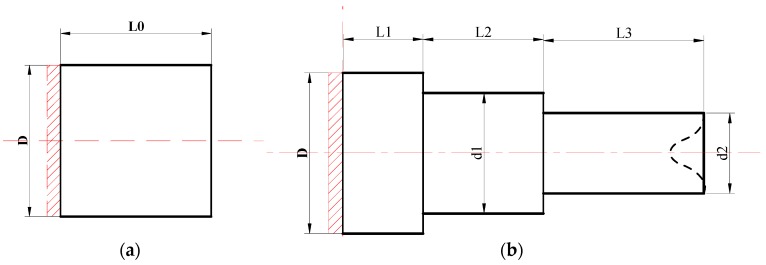
Diagrams of traditional billets and product: (**a**) traditional billet; (**b**) rolled piece.

**Figure 5:**
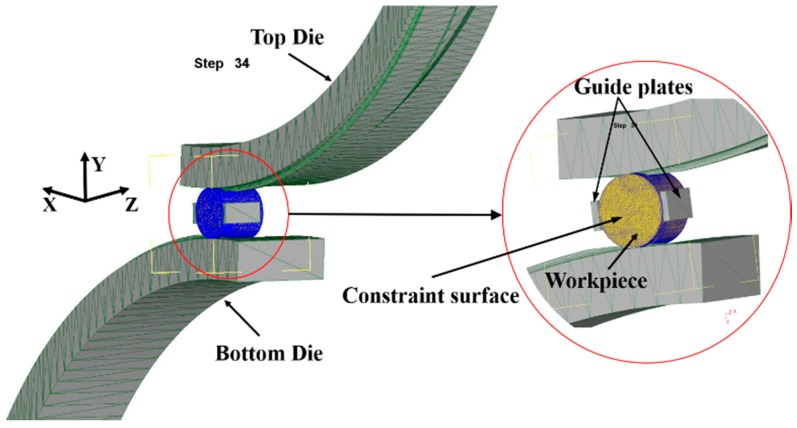
Finite element model.

The rolling process of multi-step shafts always starts from a certain plane in the middle to both ends for metal shaping and the influence of plastic metal on the concave size of the rolling end will decrease with the increasing of the distance from the shaft end [19,20]. Consequently, in order to reduce the quantity of calculation and enhance calculation efficiency, when using DEFORM-3D software for finite element simulation, a three-diameter shaft was selected as the research object, and contact surface constraint was applied to the middle of a certain plane, as shown in Figure 4. The dimension parameters of the product were as follows: D = 50 mm, L1 = 10 mm, L2 = 37.4 mm, L3 = 25.9 mm, d1 = 40 mm, d2 = 35 mm, the spreading angle of the die = 8°, forming angle = 30°, the finite element model was of 20CrMnTi material. The roller radius is 500 mm, the parameters of friction between the workpiece and rollers are 0.2, the parameters of friction between the workpiece and guide plates are 0.01, and the rolling temperature was 1050 °C. The billet was of plastic material and four-dimension mesh was applied, with the number of grid points as 150,000. The finite element model established is as shown in Figure 5.

### 2.3. Forming Features of Stepped Shaft Rolling and Forming Mechanisms of Concaves 

**Figure 6:**
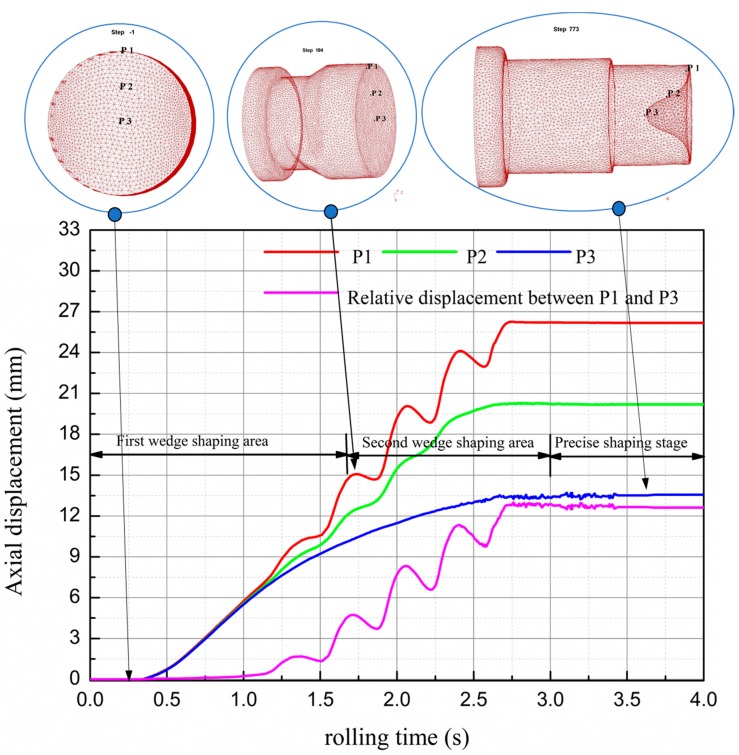
Process of forming of end concaves of stepped shaft based on the point tracing method.

The features of stepped shafts rolling are large axial and radial plastic deformation, different radial compression of metal at different stages, an uneven distribution of axial and radial deformation, etc. As a result, the end concave of a rolling piece made from stepped shaft is larger than that made from common shafts. The point tracing method was used to study the forming features of stepped shafts and the forming mechanisms of end concave. As shown in Figure 6, **P1** is at the circular edge, **P3** is at the circular center and **P2** is at the 1/2 radius of the rolling piece. The axial displacement results are as illustrated below. 

As illustrated in the figure, the forming process of stepped shaft is divided into three stages, including: the first wedge-shaping stage (0–1.71 s), the second wedge-shaping stage (1.71–3 s) and the precise shaping stage (3–4 s), and the entire process is complete within 4 s. During 0–1 s, the end metal moves axially and rises perpendicularly. During 1–2.75 s, the axial displacement velocity of **P3** gradually slows down due to the influence of the formed concave; meanwhile, **P2** and **P3** have horizontal displacement under the influence of fiber crimp and their movement path matches with the spiral curve equation. Due to the variances of deformation, **P2** at 1/2 radius is selected for calculating the movement path of the shaft end, during 0–1.71 s, the simulation result of the displacement curve’s slope is 7.2, and the theoretically calculated result is 6.9, with the error at 4%, and by 1.71–2.47 s, the simulation result of displacement curve’s slope is 8.76, and the theoretically calculated result is 9.2, with the error at 5%. Based on these results, the movement laws of end metal during stepped shaft rolling basically match with plastic flow kinetic theories. 

Based on the forming of end concave in the process of rolling, within 0–1 s, the first wedge starts to wedge into and spread the billet. At the moment, the percentage reduction of area is relatively small, and the quantity of metal deformation is not huge. As a large quantity of metal exits at the ends, metal flow is restrained to a certain extent. As a result, the overall deformation is uniform and concave does not occur. At the stable rolling stage, metal moves axially and rises perpendicularly. At 1.72 s, the second wedge shaping stage starts. At this moment, the quantity of transient metal deformation increases with increasing the ratio of reduction of area. Due to the crimping of metal fibers, the surface metal moves as per the spiral path, the axial motion of core metal is constrained, and the concave can be clearly observed at the rolling ends. This happens because the quantity of metal at the ends is less, the function of inhibition is weakened, and the flow velocity of the surface metal is obviously faster than that of the core metal. Besides, the accumulation effect of uneven deformation starts to occur. The axial displacement slope of core metal gradually reduces and that of surface metal increases on the contrary. Since 1.83 s, the second wedge spreading stage starts. At the moment, the three steps have been initially formed, and relatively deep concave can be seen at shaft ends. This is because in this stage, the radial compression is large, making the end “bowl-like”: the surface metal is thin, the core is hollow, and the end metal loses its function of inhibition. As for the end metal, the radial compression and lateral extension are extremely uneven, and the phenomena of folding occurs; as for the core, the axial flow gradually slows down, and the surface metal moves perpendicularly at the axial direction. The helix lead and amplitude of the movement path gradually become larger from the outlet to the inlet point of the deforming area, and the increasing rate of concave depth is accelerated.

**Figure 7:**
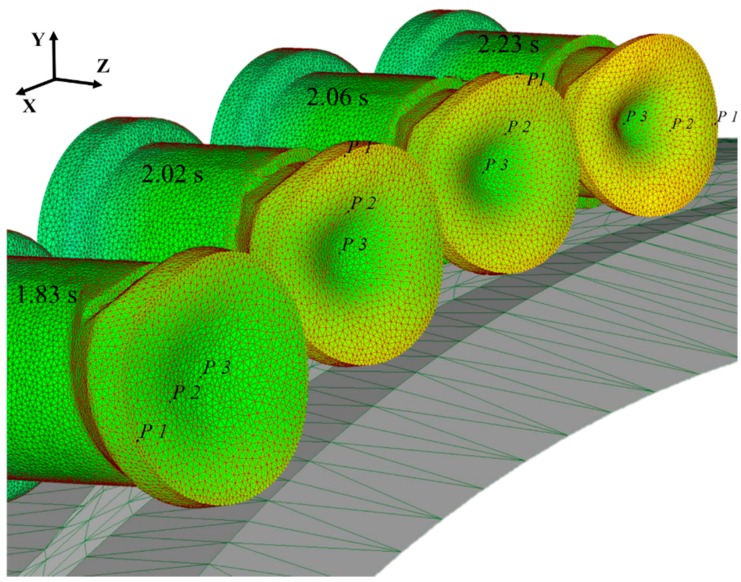
Variation of the rolling piece in the rotation period of 1.83–2.23 s.

**Figure 8:**
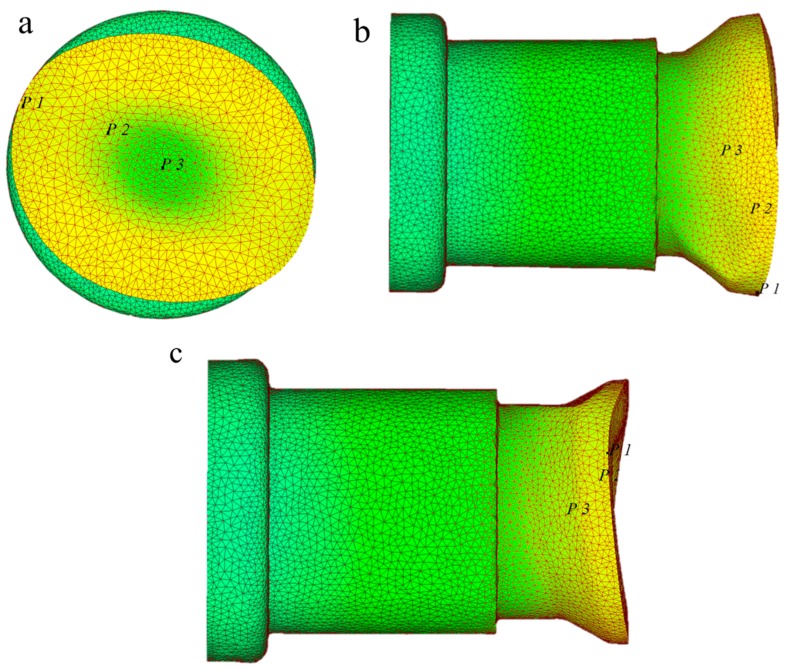
Three views of the rolled piece when rolling time is 2.23 s: (**a**) *Z* axis direction view of rolled piece; (**b**) *Y* axis direction view of rolled piece; (**c**) *X* axis direction view of rolled piece.

Take the rotation period within 1.83–2.23 s as an example. As shown in Figure 7, during 1.83–2.06 s, the roller starts to compress the **P1**–**P3** section. At the moment, the radial compression gradually becomes larger, and the surface metal starts to fold to the middle. The axial extension of the core metal is restrained, and the flow velocity slows down gradually; on the contrary, the axial extension of the surface metal is free of restraining, and the flow velocity accelerates, making the concave enlarges gradually. By 2.02 s, the roller is vertical with the **P1**–**P3** section, and radial compression reaches the peak value. However, due to the crimping of metal fibers during the CWR process, the forming of the φ angle is hysteretic, that is to say, by 2.06 s or so, the concave is the largest. Within 2.06–2.23 s, metal at the **P1**–**P3** section extends laterally, the **P1**–**P3** vertical section gradually starts its radial compression stage, and the folding effect occurs. Metal at the outer edge of the **P1**–**P3** section moves axially to the inner side, and the concave becomes smaller. The effect of folding is gradually weaker with the approaching of the core, and the core metal will not be affected. By 2.23 s, the folding effect has the most significant effect, and consequently, concave reaches its minimum value, as shown in Figure 8. 

### 2.4. Design of Billet Shape and Features of Shaft Ends Made of Billets with Variable Cone Angle 

**Figure 9:**
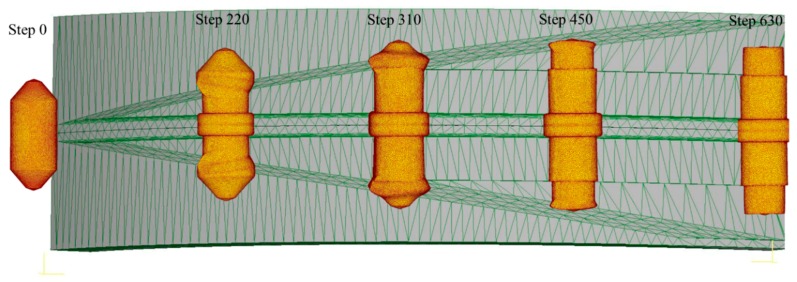
Finite element simulation of a variable cone angle billet forming to be stepped shaft by cross-wedge rolling.

**Figure 10:**
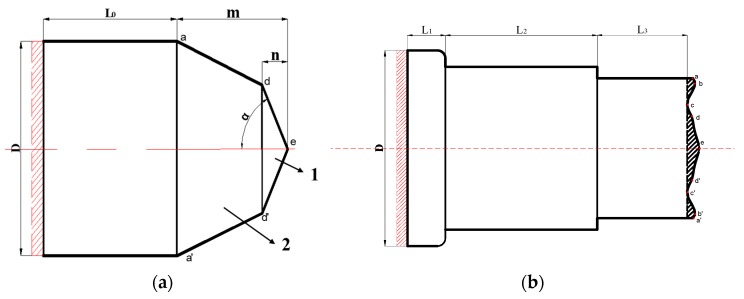
Shapes of billet with a variable cone angle and its product: (**a**) shape of billet with a variable cone angle; 1 is the first cone section, 2 is the second cone region; (**b**) shape of a typical rolling piece from such a billet.

As the rolling of stepped shafts features large metal deformation, which is in stages and uneven, the billet end is designed to the shape of the variable cone angle, to gradually weaken or even eliminate the effect of uneven axial and radial deformation, and maintain the levelness of the rolling end. The forming process of the stepped shaft by cross-wedge rolling with the variable cone angle billet is shown in Figure 9. Figure 10 shows the diagrams of the billet with the variable cone angle, and the shape of a typical rolling piece made from such a billet.

As shown in Figure 10b, the rolling end made by the billet with a variable cone angle is “W”-shaped. The depth of the concave is the distance from point **c** to **a**. This is because during the forming of a stepped shaft, the core metal of the billet will be ahead of the metal on the edge during moving. As a result, in the process of forming, hysteretic metal is between point **a** on the edge and point **e** in the core. Due to the unevenness of deformation and the crimping of metal fibers, the metal on the edge would fold along those points with the minimum displacement, coming into being the deepest area of the concave, that is, point **c**. 

In order to improve the quality of rolling ends, this paper, based on the results of single-factor tests, studied the influence laws of dimensions of the billet with a variable cone angle on the quality of end concave of the stepped shaft. The parameters and results of single-factor tests are shown in Table 1. 

## 3. Results and Discussion

### 3.1. Influence of the Total Cone Section Length m on Concave Depth 

**Figure 11:**
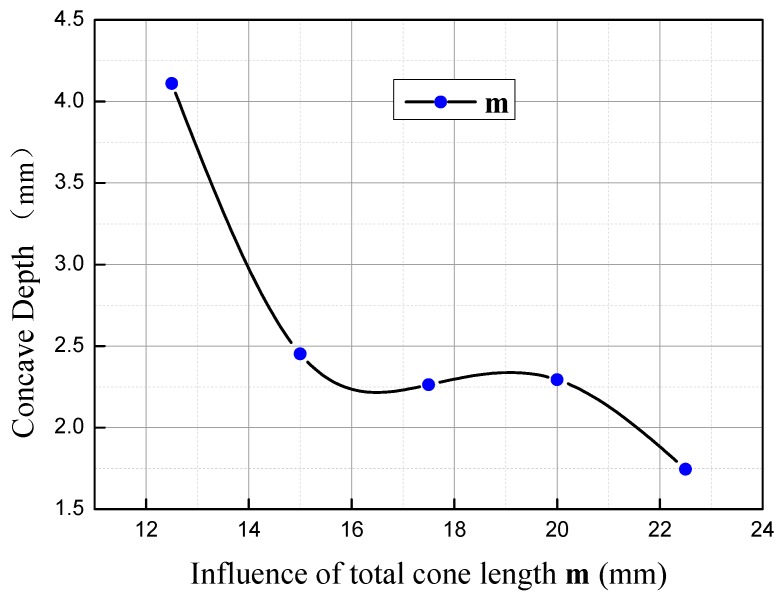
Influence of total cone length **m** on concave depth.

When **n** = 3 mm and **α** = 70°, the influence of **m** on concave depth is as shown in Figure 11. As illustrated, the depth will decrease obviously with the increasing of **m**. When **m** = 12.5–15 mm, the depth of concave changes tremendously with the increasing of **m**, in which the rate of change is −0.66. When **m** = 15–20 mm, the change of the depth with the increasing of **m** slows down, approximately at the rate of −0.03, and when **m** = 20–22.5 mm, the change is faster, with the rate increasing to −0.22. Within the range from 12.5 mm to 15 mm, **m** makes the most significant difference on concave depth, which is about three times compared to the range [20, 22.5]. 

Within the range of 12.5–17.5, when **m** = 12.5 mm, as the displacement hysteresis point **c** is close to point **d**, the depth of concave is mainly subject to the difference between the axial displacement of the metal at point **d** and those on the edge. At the beginning of rolling, as the displacement of the core metal compared to those on the edge is subtle, the axial displacement of metal within the 1/4 radius range nearby point **e** is smaller than that of the metal on the edge during the second wedge-spreading stage. As a result, point **d** is relatively hysteretic to point **a** and **e** to a certain extent, that is to say, the distribution from the core to the edge is U-shaped, and the folding effect occurs, making point **d** the core of concave. With the increasing of **m**, the second cone section becomes larger, the conicity becomes smaller, and the core metal at shaft end moves much more ahead of the metal on the edge. As a result, the axial displacement difference between the metal within the 1/4 radius range near point **e** and the metal on the edge is smaller. Meanwhile, point **c** slowly moves from point **d** to the range between point **d** and **a**, the radial distance of folding becomes shorter, the folding effect becomes weaker, and the concave is shallower. 

Within the range of **m** = 17.5–20 mm, due to the distance advantage of metal at point **e**, it is flush with point **a** at the axial direction upon the completion of rolling. With the increasing of L, as the conicity difference between the second and the first cone sections becomes larger, at the second wedge-spreading stage, the resistance, due to the core metal of the cone, against the axial flow of the metal between point **a** and **d** is gradually larger, point **c** continues to move to point **b**, the cone point angle ∠ded’ gradually becomes smaller, and the first cone section as a whole starts to move ahead of the metal on the edge. Within the range from 17.5 mm to 20 mm, due to the restriction of cone angle against metal flow, the folding effect should be more significant, and consequently, the concave depth slightly increases.

Within the range of **m** = 20–22.5 mm, with the increasing of **m**, the impediment effect of cone angle becomes larger, the hysteretic distance of point **c** increasingly becomes larger, the phenomena of folding occurs earlier, and the folding effect is more obvious. Meanwhile, with the proceeding of the rolling process, the core metal of the cone moves laterally due to the influence of hysteretic metal and torsional effect. The metal at point **c** is compensated, and the complementary effect offsets the folding effect. As a result, concave depth decreases. 

### 3.2. Influence of the Length of the First Cone Section n on Concave Depth 

**Figure 12:**
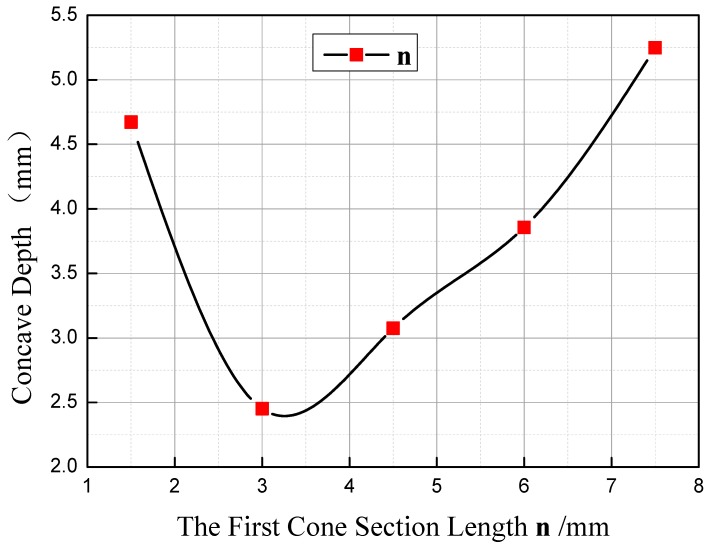
Influence of the first cone section length **n** on concave depth.

The curve of change of concave depth with **n** is as shown in Figure 12. As illustrated, when **m** =15 mm and **α** = 70°, concave depth will firstly decrease and then increase with the increasing of **n**. When **n** = 1.5–3 mm, the larger **n** is, the smaller the concave depth will be, and the change tendency is fast, with the rate at −1.48. When **n** = 3–7.5 mm, the larger **n** is, the larger the concave depth will be, and the change tendency slows down, at the rate of 0.62. 

Based on the forming mechanism of concave in stepped shaft, when **n** changes from 1.5 mm to 3 mm, the depth of concave is subject to the deformation unevenness degree of metal on the edge and between point **a** and **b**. With the increasing of **n**, the volume of the first cone section will enlarge, the difference of initial displacement between the metal in the core and that on the edge becomes larger, and the radial distance between point **a** and **d** becomes shorter. Consequently, the deformation unevenness of the metal between point **a** and **c** decreases, the axial displacement difference between point **a** and **c** becomes smaller, and the concave depth decreases accordingly. Within the range of **n** = 3–7.5 mm, the depth of the concave is subject to the deformation unevenness of the metal on the edge and that within the 1/4 radius range. At the moment, the radial distance between point **a** and **d** becomes shorter, that between point **d** and **e** becomes longer, and point **c** moves from the middle of point **a** and **d** to the middle of point **d** and **e**. With the increasing of **n**, the difference of initial displacement at the axial direction of the metal at point **d** and **e** becomes larger, the deformation unevenness of metal becomes larger, and the concave depth increases accordingly. 

### 3.3. Influence of Cone Angle α on Concave Depth 

**Figure 13:**
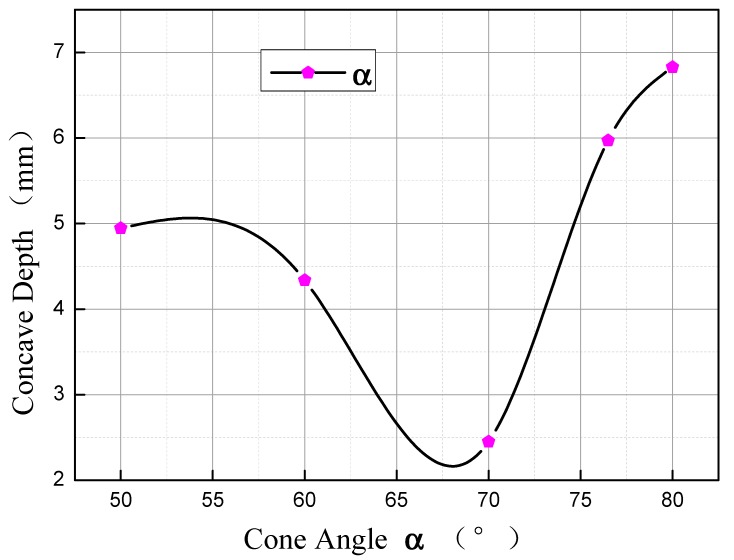
Influence of cone angle **α** on concave depth.

The curve of change of concave depth with **α** is as shown in Figure 13. As illustrated, when **m** = 15 mm and **n** = 3 mm, concave depth will firstly decrease, and then increase with the increasing of **α**. When **α** = 50–68°, the larger **α** is, the smaller the concave depth will be, with the change rate at −0.125 mm/°. When **α** = 68–80°, the larger **α** is, the larger the concave depth will be, with the change rate at 0.44 mm/°, which is about 3.5 times the change rate within the range of 50° to 68°. 

Within the range from 50° to 70°, the concave depth is subject to the deformation unevenness of metal on the edge and metal at point **d**. When **α** = 50°, point **c** and point **d** overlap. As the cone angle is small and the displacement advantage of point **e** relatively to point **d** is obvious, the concave is a U-shaped distribution from the core to the edge during rolling, and the metal will fold along point **d**. With the increasing of the angle, the volume of the first cone section increases, the difference of the initial displacement at the axial direction between point **e** and point **d** becomes gradually smaller, the flow line of metal is improved, the folding effect at point **d** gradually becomes weaker, and the concave depth becomes smaller. Within the range from 70° to 80°, the depth of concave is mainly subject to the axial displacement difference between point **d** and point **e**. With the gradual increasing of the cone angle, the initial displacement difference at the axial direction between point **d** and **e** gradually becomes smaller, the radial distance enlarges, the deformation unevenness increases, and the depth of the concave increases accordingly. 

### 3.4. Comprehensive Analysis of the Influence Degree of the Shape Parameters of Billets

**Figure 14:**
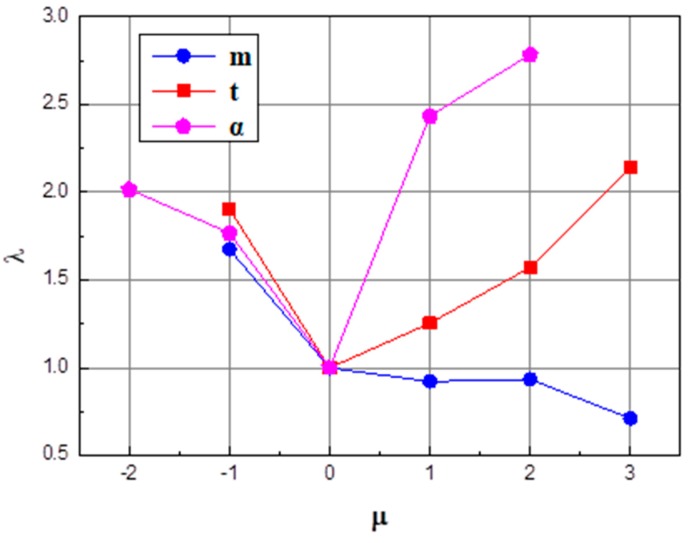
Comparison of influence of different factors on the concave quality of the rolling end.

Different shape parameters of the billet have different impacts on the quality of concave at rolling end, and each factor has a different dimension. It cannot directly compare the importance of all factors to the end quality. In order to further confirm the priority sequence thereof, influence factor **μ** and influence coefficient **λ**, both with the dimension as 1, are introduced herein. **μ** refers to the relative variation of factors, and **λ** refers to the relative variation of the concave depth. Through the analysis of these data, the influence laws of different influence factors on the quality of the shaft end are analyzed and obtained, as shown in Figure 14.

(9) $$\mu =\frac{A_{i}}{A_{0}};\;\lambda =\frac{B_{i}}{B_{0}}$$

where in *A_i_* is the level value of the influence factor, *A*_0_ is the level value of the third group of experiment factors, *B_i_* is the depth of the end concave, and *B*_0_ is the depth of the end concave of the third group of experiment. 

As illustrated in Figure 11, the parameters of cone angle **α**, the height of the first cone section **n**, and the total cone height **m** have an increasingly weaker influence on the end concaves. Specifically, core angle **α** has the most significant impact on the end concave’s quality, which is about two times the influence compared to the impact on total cone height **m**. When **μ** < 0, the difference of influence from these three parameters on concave is not obvious, so the sequence of influences from huge to small is the height of the first cone section, the cone angle, and the total cone height. When **μ** > 0, the cone angle **α** and the height of the first cone section **n** start to have a direct proportional relationship to the influence on the concave; the influence ratio of the cone angle **α** increases, and is 56% larger than that of the height of the first cone section **n**. Meanwhile, the total cone height continues to have an inverse proportional relationship to its influence ratio on the concave, but the influence level is weakened, and is about 50% of the influence within the range from −1 to 0. 

### 3.5. Experimental Verification 

**Figure 15:**
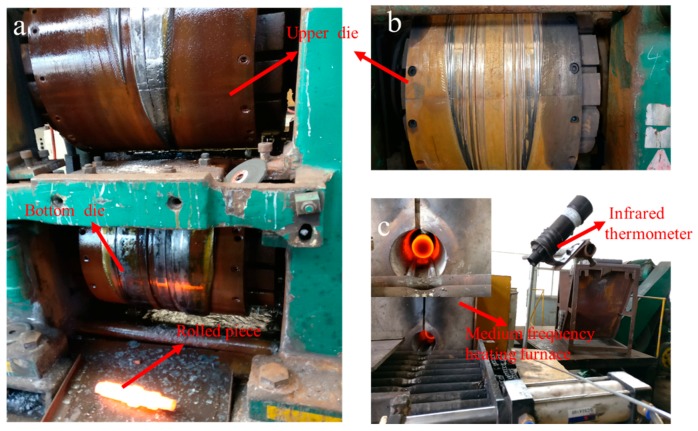
Diagram of the rolling site: (**a**) the H630 type rolling mill; (**b**) the cross-wedge rolling die; (**c**) heating device.

**Figure 16:**
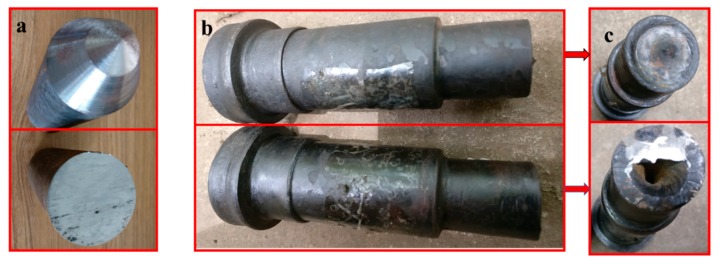
Billets and rolled products: (**a**) comparison of billets; (**b**) lateral comparison of rolled products; (**c**) axial comparison of rolled products.

**Figure 17:**
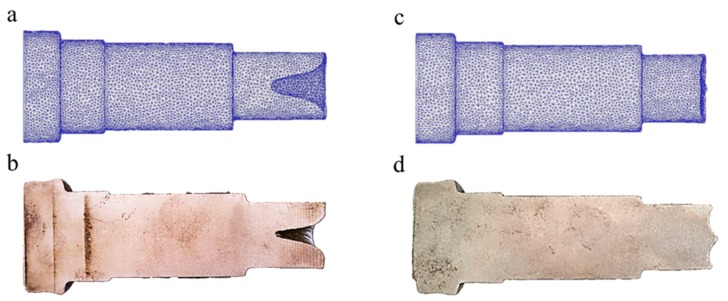
Comparison of Simulation (**a**,**c**) and Experimental (**b**,**d**) rolling effects of stepped shafts: (**a**) simulation results of rolling by a traditional billet; (**b**) experiment results of rolling by traditional billet; (**c**) simulation results of rolling by a billet with a variable cone angle; (**d**) experiment results of rolling by a billet with a variable cone angle.

Experiments were fulfilled on H630 rolling mills and Figure 15a–c shows respectively the rolling mill, rolling die, and a picture of the infrared thermometer. Stepped shaft products from a traditional billet and from a billet with a variable cone angle are shown in Figure 16. Figure 17 is the comparison between simulation and experimental results. Table 2 compares the statistical data of the simulation and experimental concaves. 

Through comparison, the simulation and experimental results are basically consistent. Specifically, the simulated concave depth using traditional billet is 25.44 mm, and in practical rolling, the figure is 24.95 mm, with the error ratio at 1.9%. By means of billets with a variable cone angle, the simulated depth is 1.93 mm, and the practical result is 1.85 mm, with the error at 4%. The depth of concave of the rolling pieces using a cone-shaped billet is about 92.5% smaller than those using traditional billets, and the utilization rate is 14% higher comparatively. Based on the results of finite element simulation and rolling experiment, the rolling of stepped shafts using billets with a variable cone angle has the outstanding impact on resolving end concave problems and enhancing the utilization rate of the material. 

The billet end can be preformed to a variable cone angle shape before cross-wedge rolling by the hot roller shearing equipment [21,22] developed by our research group, and the near-net forming technology for stepped shaft by cross-wedge rolling based on variable cone angle billets has also reached the industrial application level.

## 4. Conclusions

1. In the process of cross-wedge rolling (CWR), metal in the shaping area will crimp like fibers, and the axial movement path of the end metal is in line with the spiral curve equation. During the rolling of the stepped shaft, subject to the impact of the changes of the percentage reduction of area, fiber crimp of metal, etc., the shaping process of the concave develops by stages, and the depth is increased by the wave mode. The adoption of a billet with a variable cone angle can effectively enhance the quality of stepped shaft ends and increase the utilization rate to more than 99%.
2. The parameters of the cone angle **α**, the first cone section length **n**, and the total cone section length **m** have increasingly weaker influence on end concaves. Specifically, the cone angle **α** makes the most significant impact on the end concave’s quality, which is about two times the influence of the total cone section length **m**. The depth of concave will firstly decrease and then increase with the increasing of the cone angle **α** and of the first cone section length **n**, and will decrease with the increasing of the total cone section length **m**.
3. Through comparison, the error ratio between the experimental and simulation results of rolling stepped shafts using billets with a variable cone angle is lower than 5%. It means that the results of finite element simulation are reliable, and can reflect the changes of shaping of the rolling piece during practical production.

## Figures and Tables

**Table 1:** Test design and results of single-factor test.

Number	m/mm	α/°	n/mm	Concave Depth/mm
1	15	50	3	4.946
2	15	60	3	4.337
3	15	70	3	2.452
4	15	76.5	3	5.972
5	15	80	3	6.828
6	15	70	1.5	4.672
7	15	70	3	2.452
8	15	70	4.5	3.075
9	15	70	6	3.856
10	15	70	7.5	5.249
11	12.5	70	3	4.111
12	15	70	3	2.452
13	17.5	70	3	2.263
14	20	70	3	2.294
15	22.5	70	3	1.745

**Table 2:** Concave depth statistics of finite element simulation and experimental results of stepped shaft by cross-wedge rolling.

	Concave Depth	Finite Element Simulation Results/mm	Experimental Results/mm	Material Utilization
Billet Type				
traditional billet		25.44 mm	24.95 mm	86.8%
billet with variable cone angle		1.93 mm	1.85 mm	99%
